# Neuroinspired unsupervised learning and pruning with subquantum CBRAM arrays

**DOI:** 10.1038/s41467-018-07682-0

**Published:** 2018-12-14

**Authors:** Yuhan Shi, Leon Nguyen, Sangheon Oh, Xin Liu, Foroozan Koushan, John R. Jameson, Duygu Kuzum

**Affiliations:** 1 Department of Electrical and Computer Engineering, University of California, San Diego, La Jolla, CA 92093 USA; 2 Adesto Technologies Corporation, 3600 Peterson Way, Santa Clara, CA 95054 USA

## Abstract

Resistive RAM crossbar arrays offer an attractive solution to minimize off-chip data transfer and parallelize on-chip computations for neural networks. Here, we report a hardware/software co-design approach based on low energy subquantum conductive bridging RAM (CBRAM®) devices and a network pruning technique to reduce network level energy consumption. First, we demonstrate low energy subquantum CBRAM devices exhibiting gradual switching characteristics important for implementing weight updates in hardware during unsupervised learning. Then we develop a network pruning algorithm that can be employed during training, different from previous network pruning approaches applied for inference only. Using a 512 kbit subquantum CBRAM array, we experimentally demonstrate high recognition accuracy on the MNIST dataset for digital implementation of unsupervised learning. Our hardware/software co-design approach can pave the way towards resistive memory based neuro-inspired systems that can autonomously learn and process information in power-limited settings.

## Abstract (web summary)

To realize the potential of resistive RAM crossbar arrays as platforms for neuromorphic computing, reduced network-level energy consumption must be achieved. Here, the authors use a hardware/software co-design approach to realize reduced energy consumption during network training for the network.

## Introduction

Inspired by the biological neural networks giving rise to human intelligence, artificial neural networks^1^ have revolutionized numerous computer vision^2,3^ and speech recognition^4,5^ tasks. Their near-human performance has been widely leveraged in various applications, including automated systems^6^, aerospace and defense^7^, health care^8^, and home assistance devices^9^. However, training of neural networks requires substantial computing power and time due to the iterative updates of massive number of network parameters. For example, today’s advanced neural network algorithms require training times ranging from days to weeks and use carefully organized datasets consisting of millions of images to recognize objects such as animals or vehicles^10–12^, while it only takes a few repetitions for a 2-year-old toddler to identify these accurately and effortlessly^13^. Another example is AlphaGo, an advanced neural network trained for playing the board game Go against world champions, requiring 1920 CPUs and 280 GPUs and consuming hundreds of kilowatts per game^14^. The human brain, which can perform the exact same task, is 30,000 times more efficient, only consuming power on the order of 10W^13,15^. High energy consumption and extensive training time have been the major limitations for widespread adoption of neural networks at every scale—from mobile devices to data centers. The need for back and forth data transfer between the memory and processor in conventional computing systems based on von Neumann architecture is one of the major causes of high energy consumption during neural network computations. To address this major architectural drawback, on-chip memory storage and in-memory computing solutions using resistive switching memory arrays have been proposed to perform storage and computing at the same location. Non-volatile memory-based synaptic devices such as phase change synapses (PCM)^16,17^, Ag-based conductive bridging synapses (CBRAM)^18^, and resistive RAM synapses (RRAM)^19–21^ have been investigated for implementing synaptic weight updates during neural network operation. The synaptic arrays using memristors have also been widely used in energy efficient implementation of unsupervised learning^22–25^ and MNIST classification^26–34^ in the past.

On a separate front, the pruning algorithm^35,36^ inspired from neuroscience^37^ has been suggested toward reducing network level energy consumption and time by settings the low valued weights to zero. However, these methods were mostly applied on the trained networks^35,36^. Pruning during training by backpropagation was previously employed in literature to prevent overfittings^38,39^. Yet, there is no systematic study showing how pruning can address the energy consumption and excessive training time problems during the training in hardware.

In order to overcome the energy consumption challenge, incremental improvements in devices or algorithms alone will not be sufficient. Therefore, in this work, we focus on a hardware/software co-design approach that combines the advances in low-power device technologies with algorithmic methods to reduce the energy consumption during neural network training. First, we experimentally investigate and characterize the gradual conductance change characteristics of subquantum CBRAM devices, targeting implementation of neural network training in hardware. We show that the subquantum CBRAM devices can achieve gradual switching using stepwise programming and they can be directly programmed into any arbitrary level by controlling wordline (WL) voltage. Then we develop a spiking neural network (SNN) model for unsupervised learning and evaluate its performance by simulations for both analog and digital hardware implementations. In order to improve network level efficiency, we introduce a pruning algorithm carried out during the training and investigate its limits and performance through software simulations. Different from previous algorithmic approaches employing pruning on already trained networks^35,36^, our neuro-inspired pruning method is applied during the network training to minimize the energy consumption and training time. Combining the energy-efficient subquantum CBRAM devices and the pruning technique, we experimentally demonstrate highly energy efficient unsupervised learning using a large-scale (512 kbit) subquantum CBRAM array. The hardware/software co-design approach presented in this work can open up new avenues for applications of unsupervised learning on low-power and memory-limited hardware platforms.

## Results

### Subquantum synaptic device characteristics

**Fig. 1:**
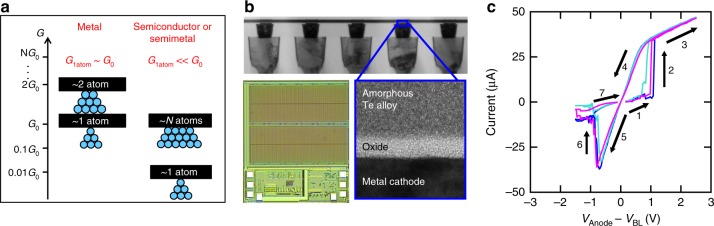
Subquantum CBRAM characteristics. **a** Semiconductor or semimetal filaments can yield lower conductance than metal filaments of comparable width. **b** Subquantum conductive bridging RAM (CBRAM) cell fabricated in a standard 130 nm logic process. Photograph shows 512 kbit subquantum CBRAM chip with one-transistor one-resistor (1T1R) array architecture. Cell cross-section shows amorphous Te alloy as anode, metal as cathode and oxide as switching layer. **c** Example of bipolar current-voltage characteristic of a subquantum CBRAM cell. Directionality of switching is shown in arrows

In this section, we investigate device characteristics of subquantum CBRAM relevant to the general context of neural network operation. We explore gradual switching capability of subquantum CBRAM for implementation of different biological or non-biological weight update rules. For CBRAM devices, the 1-atom conductance (*G*_1atom_), which corresponds to the conductance (*G*) of a filament just one atom “wide” at its thinnest point, is a critical parameter affecting energy consumption and filament stability (retention)^40^. *G*_1atom_ is on the order of the fundamental conductance *G*_0_ = 2*e*^2^/*h* ≈ 80 μS for CBRAM cells based on filament metals such as Ag and Cu, so typical programming voltages of about 1–3 V yield a minimum programming current (i.e., to form a filament just 1-atom “wide”) of *I*_prog_ ≈ *G*_0_(1–3 V) = 80–240 µA, resulting in high energy consumption in the range from about 1 to 100 pJ for commonly used programming pulse durations (10–100 ns) (Supplementary Table 1). Subquantum CBRAM cells reduce programming energy and improve filament stability (Fig. 1a) by utilizing filaments comprising a semiconductor or semimetal (at least at their thinnest spot, which dominates the resistance)^40^. A subquantum CBRAM memory cell utilizing tellurium (Te), an elemental semiconductor with a band gap of 0.3 eV^41^, which has a 1-atom conductance deduced^40^ to be *G*_1atom_ = 0.03*G*_0_, is shown in Fig. 1b. With a much lower *G*_1atom_ than Ag or Cu and with write/erase speeds as low as about 10 ns (Supplementary Figure 1), such subquantum CBRAM cells can consume as little as about 0.2 pJ (*I*_prog_ ≈ 0.03*G*_0_(1–3 V) ≈ 2.4–7 µA and *E* = *I*_prog_ × *V*_prog_ × pulse duration = 7 µA × 3 V × 10 ns = 0.2 pJ) when programmed to their 1-atom limit. This is an order of magnitude lower than for metal filament-based devices programmed to their corresponding 1-atom limit (Supplementary Table 1). The retention of the subquantum CBRAM device is shown in Supplementary Figure 2 and is discussed in Supplementary Note 1.

Figure 1b shows a cross-section TEM of a subquantum CBRAM cell, fabricated using Ta as the cathode material, sputtered amorphous Al_2_O_3_ as the insulating layer, and sputtered amorphous ZrTe as the anode material. The array (Fig. 1b) containing the subquantum CBRAM device has one-transistor one-resistor (1T1R) structure, which provides access to individual cells. I–V characteristics of subquantum CBRAM cells measured by a typical double DC sweep exhibit bipolar characteristics (Fig. 1c). In the positive regime, a voltage bias is applied to the anode and swept from 0 to + 3 V with step size 5 mV. The resistance of the cell was switched from a high resistance state to a low resistance ON-state. This process is suggested^40^ as inducing an electrochemical replacement reaction wherein Te is liberated from the anode by O from the oxide layer. In the negative regime, reversing the polarity of the voltage will break the filament and switch the cell back to a high resistance OFF-state. The resistance can be read without disturbing the state of the cell by applying a small voltage (~100 mV) of either polarity. These two distinct states are utilized in memory applications to store binary information. On the other hand, a gradual, analog-like conductance change has been suggested as a requirement for implementation of synaptic plasticity and learning^42^. Gradually increasing and decreasing device conductance is equivalent to long-term potentiation (LTP) and long-term depression (LTD) of synapses in the brain, which are two major forms of synaptic plasticity. LTP and LTD allow for fine synaptic weight updates during network training. Subquantum CBRAM cells can potentially provide more gradual changes in conductance than metal filament-based cells since during programming G tends to increase in increments of ~*G*_1atom_, which for Te is an order of magnitude smaller than for metals.

**Fig. 2:**
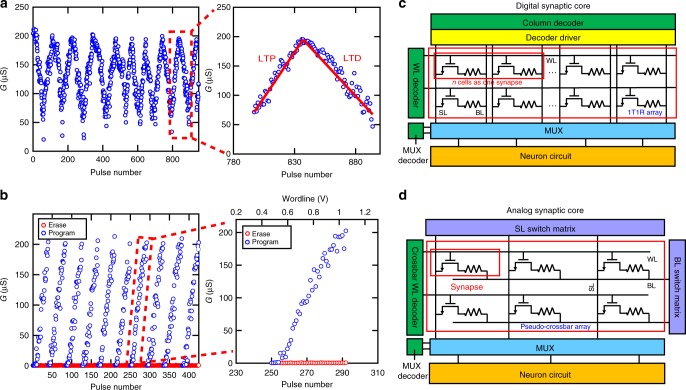
Gradual switching. **a** Gradual switching in a subquantum conductive bridging RAM (CBRAM) synapse using stepwise voltage pulses applied to the wordline (WL) (left). Callout window (right) shows one cycle of long-term potentiation (LTP) and long-term depression (LTD). Red lines are added to emphasize linearity of the conductance change. For LTP, anode (AN) = 3 V, bitline (BL) = 0, and WL stepped from 0.8 V in increments of 10 mV. For LTD, AN = 0, BL = WL, and WL stepped from 1.6 V in increments of 4 mV. **b** Gradual switching in a subquantum CBRAM synapse by WL voltage modulation. The subquantum CBRAM cells are directly programmed into the conductance state by controlling the WL voltage. The figure (left) shows a sequence of programming operations in which the WL voltage increases with step size 20 mV followed each time by an erase operation. Callout window (right) shows conductance versus pulse number and WL voltage for a representative cycle. **c** Digital synaptic core design groups multiple binary one-transistor one-resistor (1T1R) cells along the row as one synapse to represent a synaptic weight with higher precision. WL decoder is used to activate the WL in a row-by-row fashion. Column decoder can select a group of synapses to perform the weight update. The weighted sum is implemented using mux and neuron circuit. The mux is used to share the read periphery circuitry^46^. The neuron circuit which contains sense amplifier, adder and shift register can be used to read out the memory array and accumulate partial weight sum to get the final weighted sum. **d** Analog synaptic core uses a single cell with multi-level conductance states to represent one synaptic weight. The crossbar WL decoder can activate all WLs, BL read out the weighted sum results and neuron circuit contains analog-to-digital (ADC) converters convert current to digital outputs. Source line (SL) can be used to perform weight update^46^

We investigate general gradual programming characteristics of subquantum CBRAM cells using two different methods. Controlling WL voltage allows to change programming current values to program the CBRAM devices to different conductance levels, as this property of resistive memories has been studied before. Figure 2a shows gradual switching of a subquantum CBRAM cell by application of stepwise voltage pulses applied to the WL with an increasing step of 10 mV for conductance increase and 4 mV for conductance decrease over many cycles. Subquantum CBRAM cells can provide linear weight tuning for both LTP and LTD (Fig. 2a, as shown by linear trend lines). The linearity of the weight tuning was previously reported to be important for implementation of various operations and achieving high accuracy in artificial neural network implementations with resistive memory devices^43,44^. Stepwise gradual programming of subquantum CBRAM synapses (Fig. 2a) can be used to`implement various forms of learning and plasticity. As representative examples, Supplementary Figure 3 shows two different forms of biological spike-timing-dependent plasticity (STDP)^16,42,45^ implemented with subquantum CBRAM synapses. Symmetric plasticity (Supplementary Figure 3a) can be employed for associative learning and recall^16^, and asymmetric plasticity (Supplementary Figure 3b) can be used to transform temporal information into spatial information for sequence learning^16^. The STDP implementation is discussed in Supplementary Note 2.

Alternative to stepwise programming, the subquantum CBRAM cells can also be directly programmed into an arbitrary conductance state by controlling the WL voltage without being bound to a particular sequence of states. Figure 2b shows a sequence of programming operations in which the WL voltage increases with step size 20 mV followed each time by an erase operation. This offers flexibility for implementing weight update rules of greater complexity. Supplementary Figure 4 shows that the nonlinear weight update rule we used can be greatly represented by the device conductance change using this WL voltage modulation.

In order to implement neural network training with 1T1R resistive memory arrays, synaptic weights can be represented in either binary (digital) or analog manners^46^. For digital implementation, N binary 1T1R cells are grouped to represent one synaptic weight (Fig. 2c) and each cell is programmed to high or low conductance states, providing N-bit weight precision in a binary format. For analog implementation, the cells can be arranged into a pseudo-crossbar array and synaptic weights are stored in the form of multi-level conductances (Fig. 2d)^46^. As shown in the measurement results presented in this section, the subquantum CBRAM devices are capable of both digital and analog implementations. The tradeoff between analog and digital implementations in terms of energy consumption, latency and area will be further discussed in the context of our neural network model in the following section.

### Neural network algorithm for unsupervised learning

**Fig. 3:**
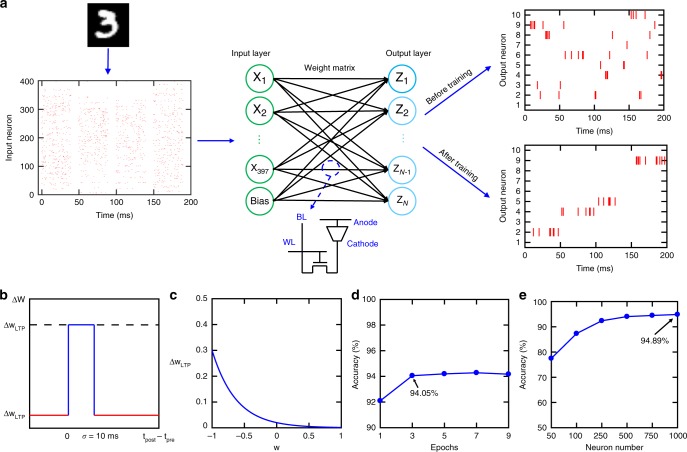
Neural Network for unsupervised learning. **a** Each input digit contains 28 × 28 = 784 pixels and has been cropped and reduced to 397 pixels. The neural network has 397 input neurons with a bias term and 500 output neurons. Input spike trains of input neurons are generated according to pixel density (from 0 to 1) and then fed to the neural network. Synaptic devices represent weights in the network. Top (before training): Random spike activity from representative 10 out of 500 output neurons before learning. Bottom (after training): Output spike trains after learning show coordinated selective firing activity as a result of unsupervised learning of digits. **b** Spike-timing-dependent plasticity (STDP) rule showing the 10 ms window for an post-pre spike time difference (*t*_post_ − *t*_pre_) that determines whether a long-term potentiation (LTP) or a long-term depression (LTD) update is performed. If the firing time of an output neuron (*t*_post_) is within 10 ms of the firing time of an input neuron (*t*_pre_), the weight (synapse) between this input–output neuron pair is updated via LTP. Otherwise, the weight is updated via LTD. **c** The LTP update is an exponentially decaying function that depends on the current weight, and the LTD update is a constant. The exponential LTP update depending on the current weight keeps the weight values within the range [−1, 1]. **d** Recognition accuracy vs. number of training epochs. Three epochs are used in our network training. **e** Recognition accuracy vs. neuron number. Recognition accuracy does not have noticeable increase when number of output neurons is larger than 500. Therefore, 500 output neurons are used in our network model

Here, we investigate neuro-inspired SNN configurations and implement unsupervised learning on 1T1R CBRAM synaptic arrays to classify MNIST handwritten digits, which consists of 60,000 training samples and 10,000 test samples. Different from other neural networks trained using backpropagation, neuro-inspired SNNs use event-based and data-driven updates to reduce redundant information processing to gain efficiency and minimize energy consumption, making them ideal for hardware implementations^47–49^. Neuromorphic hardware platforms based on SNNs have already been demonstrated and employed in various applications of neural networks^48–50^. To reduce the network size, we crop some black background pixels from the full image of 784 (28 × 28) pixels. Therefore, our network contains 397 input neurons with a bias term and 500 output neurons, resulting in 199,000 synaptic weights (Fig. 3a). SNNs encode information between input and output neurons using spike trains. The firing frequency of the Poisson spike trains generated by the input neurons scales linearly with respect to the pixel intensity (0 Hz for intensity value of 0 and 200 Hz for intensity value of 1). The output neurons integrate all the inputs to generate output spike trains based on a probabilistic winner-take-all (WTA) mechanism (see Methods section for more details)^51,52^. The synaptic weights of the firing output neuron are updated by a simplified STDP rule shown in Fig. 3b during training. STDP rule that modulates weights based on the timing of input and output spikes: if the time difference between the post-spike and pre-spike is <10 ms, the synaptic weight is updated via the LTP rule, otherwise, it is updated via the LTD rule. Here, the LTD update is a constant weight decrease and the LTP update depends on the current weight state of the synapse with an exponentially decaying function shown in Fig. 3c. Exponential LTP updates will guarantee that the weights converge to the upper bound of 1. For LTD updates, the lower bound of the weight is clipped to −1. Overall, these rules result in weight values that are in the range of −1 to 1, allowing for a feasible and practical hardware implementation. During the training, the weights are adjusted incrementally based on the STDP rule so that output neurons fire selectively for a certain class in the dataset. Before training, output neurons exhibit random spiking response to the presented digits (Fig. 3a). However, after training, output neurons fire selectively during the presentation of specific samples learned during the training (Fig. 3a). Figure 3d and e show MNIST digit classification accuracy as a function of training epoch and neuron number. Training more than 3 epochs (Fig. 3d) or increasing the output neuron number beyond 500 (Fig. 3e) do not result in noticeable increase in accuracy, similar to what has been reported for single layer spiking neural networks in literature^53^. Therefore, we choose to use 500 neurons and 3 epochs for the training in our analysis. The algorithm we used for unsupervised learning is summarized in Supplementary Figure 5. After training is complete, the training dataset is presented again to assign neuron labels to the output neurons by determining which digits provoked the highest average firing rate for each of the output neurons^53^. We predict the labels from the test set, which consists of 10,000 new samples from the MNIST test set, based on the same framework used during training to find the output neuron with the highest average firing rate for each sample (see Methods section for more details). We simulate our network for the ideal software (64-bit), and our proposed digital (Fig. 2c) and analog implementations (Fig. 2d). Table 1 summarizes classification accuracy for all three cases. For the ideal software implementation, it is important to point out that ~94% accuracy is already very high for unsupervised learning with SNN^53^. Increasing the accuracy further to the levels of deep neural networks will definitely require introducing supervision to the SNN^54–56^. For digital implementation, we use 8-bit digital synapses and the weights are quantized to 256 levels distributed evenly between [−1, 1–2/256]. For analog implementation, we directly use conductance values (Fig. 2a) from device characteristic in our simulation to perform weight update during training. Neural network weights in the range of [−1, 1] can be mapped to device conductance using a linear transformation, as explained in the Methods section. Our results suggest that 8-bit digital implementation achieves comparable recognition accuracies with ideal software case and analog implementation has slightly lower accuracy due to the limited conductance states exhibited by each CBRAM synapse.

**Table 1:** Network accuracy

Precision	Accuracy
64-bit	94.05%
CBRAM (analog)	82%
8-bit (digital)	92.02%

The table summarizes the recognition accuracy of 64-bit ideal software simulation, 8-bit digital implementation and analog CBRAM synapses implementation evaluated using our network

In order to compare the digital (Fig. 2c) and analog synaptic core (Fig. 2d), we develop a SNN platform for NeuroSim^46^ (SNN + NeuroSim). NeuroSim is a C + + based simulator with hierarchical organization starting from experimental device data and extending to array architectures with peripheral circuit modules and algorithm-level neural network models^46^. We use SNN + NeuroSim to perform circuit-level simulations (Table 2) to estimate the energy, latency and area for the digital and analog implementations using the experimental data measured from subquantum CBRAM devices (Fig. 2). The left two columns of Table 2 show benchmarking results for analog synaptic core and 6-bit digital synaptic core. 6-bit precision is chosen to match the number of levels that can be achieved by gradual programing of subquantum CBRAM devices for the analog implementation. However, in order to achieve a recognition accuracy above 90%, 8-bit precision is required. Therefore, we include the third column, showing the results for 8-bit digital case, which is also used in the hardware demonstration (see the section 'Hardware demonstration of pruning during training'). The best performing metrics are shown by^b^ in Table 2. As shown in the table, the 6-bit digital scheme has better accuracy, shorter latency and lower energy consumption. On the other hand, the analog scheme occupies smaller chip area. Therefore, the benchmarking results suggest that digital implementation could be more advantageous in terms of energy consumption and latency for hardware implementation of on-line learning using subquantum CBRAM array.

**Table 2:** Circuit-level benchmark results

	Analog	Digital (6-bit)	Digital (8-bit)
Conductance levels	57 levels (~6 bit)	64 levels	256 levels
LTP pulse	0.8−1.32 V/10 mV/1 μs	2 V/1 μs	2 V/1 μs
LTD pulse	1.6−1.84 V/4 mV/10 μs	2 V/1 μs	2 V/1 μs
Accuracy^a^	82%	85.87%^b^	92.02%
Area (µm^2^)	12,277.05^b^	35,397.34	47,233.8
Latency^a^ (s)	516	129.72^b^	401.1
Energy^a^ (mJ)	149.4097	62.911^b^	151.977
Leakage power (μW)	53.78	54.14	58.99

^a^For 60,000 training images

^b^Best performing metrics

The table summarizes circuit-level benchmark results using SNN+NeuroSim for analog synaptic core and digital synaptic core with 6-bit and 8-bit. The simulations are performed for 14 nm technology node

### Pruning during the training

**Fig. 4:**
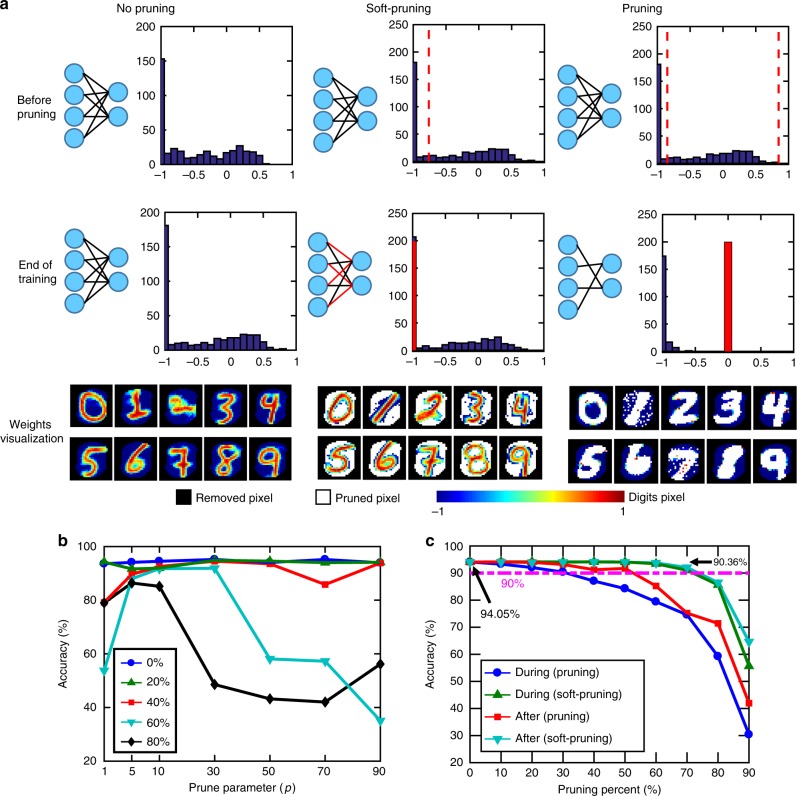
Network pruning during training. **a** Schematics compare no pruning, soft-pruning and pruning cases. Top two row shows weight histograms of a representative output neuron. For no pruning, the spike-timing-dependent plasticity (STDP) rule results in weights ranging from −1 to 1 at the end of training. For 50% soft-pruning, it prunes weights smaller than the dashed line (weights on the left of the dashed line) to the lowest value −1. 50% Pruning prunes the weights between the two dashed lines, which represent the 50% of the weights that are centered around 0 and sets their values to 0 (red bar). Only unpruned weights continue to be updated until end of training. Bottom row shows weight visualization of all representative 10 out of 500 output neurons for no pruning, 50% soft-pruning and pruning. Soft-pruning allows for the weights to still learn the foreground and background pattern of the input samples while reducing weight update computations during training. Pruning causes the pruned weights to overwhelm the learned weights and results in inaccuracy. **b** Recognition accuracy vs. prune parameter (*p*) for varying pruning percentages. Prune parameter is the criterion to decide when to prune for each neuron during training. **c** Recognition accuracy vs. pruning percentage for soft-pruning and pruning performed during training. Soft-pruning during the training performs significantly better than pruning especially for high pruning percentages. The baseline accuracy (no pruning) is 94.05%. The data points are taken in steps of 10%. The parameters used in the simulation are specified in Supplementary Table 6

Neural network pruning algorithms have been very effective to reduce the time and energy consumption during inference by removing unimportant weights. Conventional pruning methods^35,36^, which we also refer to as pruning in this work, set the low valued weights to zero. However, these methods are not suitable to be directly applied to the network learning algorithms that can produce non-zero centered weight distributions. In such situations, zero-valued weights are also important so that arbitrarily setting pruned weights to zero may affect accuracy. Additionally, conventional pruning mostly targets the networks which have already been trained. Therefore, the issues of excessive time and energy consumption during training remain unaddressed. To address both of these, we develop a method as an extension of pruning, which we refer to as soft-pruning^57^. Instead of completely removing the weights from network by setting them to zero, soft-pruning sets the values of pruned weights to a constant non-zero value and prevents them from being updated during the rest of the training while allowing them to still participate in the inference step after the training. Therefore, pruning weights during training helps to significantly reduce the number of weight updates, minimizing computation, and energy consumption. To decide when to prune weights during the training, we determine if the output neurons are trained enough to recognize a class from the dataset. We quantify this by counting the occurrences of consecutive output spikes (Supplementary Figure 6) from a single output neuron. The corresponding time interval between consecutive output spikes follows a Poisson distribution. Once an output neuron sees p occurrences of consecutive spikes during the training, a certain percentage of its weights are pruned to their lowest possible value (in our case, *W*_min_ = −1). The pruning algorithm is summarized in Supplementary Figure 7. Potential hardware implementations of this pruning algorithm are discussed in Supplementary Note 3 and associated overheads estimation in area, energy and latency via simulation (SNN+NeuroSim) are shown in Supplementary Figure 8 and Supplementary Table 2. We investigate the distribution of weights in the SNN before and after soft-pruning along with a baseline control case, where pruning is not employed (no pruning) (Fig. 4a). Simulation of recognition accuracy for different *p* values in Fig. 4b suggests that *p* = 10 provides the highest accuracy even for very large pruning percentages (up to 80%). Visualization of weights from ten representative output neurons (bottom row of Fig. 4a) shows that foreground pixels (the digits) correspond to higher weight values on the distributions, and background pixels (background of the digits) correspond to lower weight values for no pruning case (weights visualization for all output neurons can be found in Supplementary Figure 9). The Supplementary Movie 1 and 2 show the development of the output neurons’ weights during the training for both soft-pruning and no pruning cases. Before pruning, the distributions indicate that the weight updates have been the same for both cases. Figure 4c compares recognition accuracy for as a function of pruning percentage for soft-pruning and pruning during the training, in comparison to pruning at the end of training for both cases. The recognition accuracy for pruning falls below ~90% for ~40% pruning percentage. In contrast, soft-pruning maintains high classification accuracy (~90%) even up to ~75% pruning percentage (Fig. 4c). The accuracy improvement achieved by the soft-pruning algorithm can be understood from the following two perspectives. First, since the pruned weights are set to −1 instead of being completely removed from the network, they still participate in the inference. Pruning the unimportant weight to −1 effectively decreases the membrane potential of output neurons, which helps to prevent false positive spikes. Second, the soft-pruning algorithm preserves the original weight distribution. As shown in Fig. 4a, the final distribution of learned weights clearly consists of two distinct parts which correspond to the foreground and background pixels of the image. The weights concentrated at −1 are associated with the background pixels, while the remaining weights centered around zero accounts for the foreground pixels. Soft-pruning sets pruned weights to −1, grouping them with the background pixels. On the contrary, pruning sets pruned weights to 0, which is in the range of weights that are associated with foreground pixels; this significantly changes the shape of foreground weight distributions, which leads to the accuracy degradation. Our soft-pruning method achieves high recognition accuracy for extensively pruned networks, offering superior energy efficiency during training for hardware implementations of unsupervised learning.

### Hardware demonstration of pruning during training

**Fig. 5:**
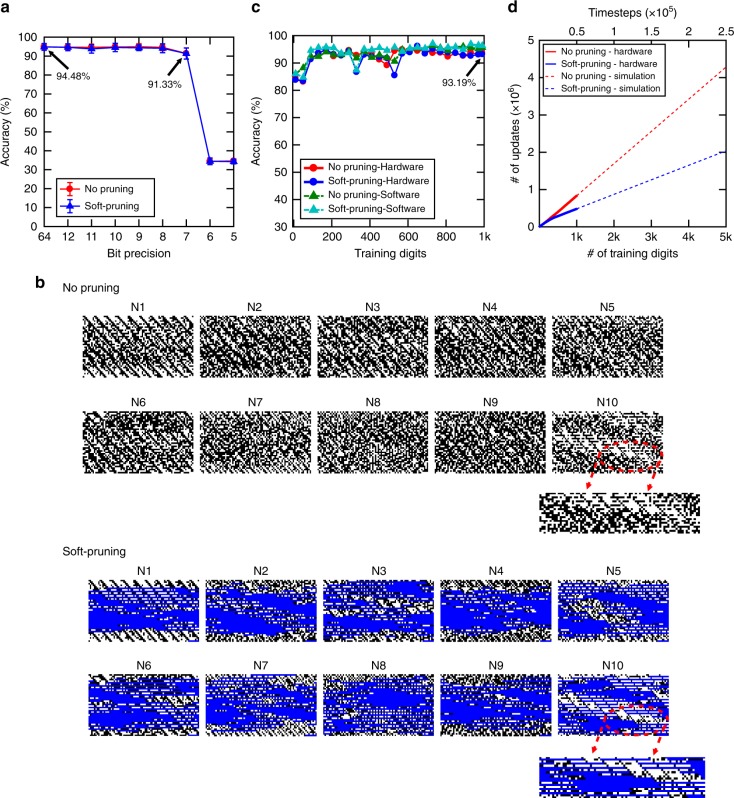
Hardware implementation of unsupervised learning and pruning. **a**, Recognition accuracy vs. bit precision. The bit precision levels include 1 bit for representing the sign. 64 corresponds to 64-bit floating point. The accuracy drops below 90% after 8 bits. Test dataset has 10k images. **b** Experimentally measured binary weights from subquantum conductive bridging RAM (CBRAM) synaptic array as a result of training with 1k MNIST digits. Binary weight 1 corresponds to black pixel, which is high resistance state (~1 MΩ). Binary weight 0 corresponds to white pixel, which is low resistance state (~10 kΩ). The bit precision per weight is 8 bits with one bit used for the sign (+/−). During the training, there is a total of 8959 weight updating events for output neurons. For no pruning (top), there were 833,889-bit updates. For training with soft-pruning at a 50% pruning rate (bottom), there were 481,921-bit updates. During the training, weights of different neurons are pruned at different times based on their learning level. At the end of training, all 10 neurons’ weights have been pruned. Bits corresponding to pruned weights are marked in blue. **c** Recognition accuracy vs. training digits for 50% soft-pruning and no pruning calculated using experimental data from hardware implementation of unsupervised learning with subquantum CBRAM array. The accuracy for pruning is comparable to no pruning. Test dataset has 10k images. **d** Number of bit updates by device updates vs. training digits/timesteps with a 50% pruning rate (blue) and without pruning (red). First 1k samples are from hardware implementation of spiking neural network (SNN) using CBRAM array

In order to implement unsupervised learning and pruning during the training on the hardware, we used a 512kbit subquantum CBRAM chip fabricated in a 130 nm Cu back end of line (BEOL) process (Fig. 1b). The array has a 1T1R architecture, which provides access to individual cells. Although each individual cell in our array has gradual conductance switching capabilities as demonstrated in Fig. 2a and b, the digital implementation offers smaller energy consumption and shorter latency which is important for online learning as shown in Table 2. Furthermore, analog approach with varying amplitude pulses requires peripheral neuron circuits to produce non-identical pulses with fine grained duration^58,59^. Therefore, we choose to use digital implementation for hardware demonstration. We uniformly quantize the weights and map them onto the CBRAM array using an 8-bit digital representation between *W*_min_ = −1 and *W*_max_ = 1 (details are explained in the Methods section), as our simulations have shown high recognition accuracy for 8-bit representation. Each weight is approximated to its closest quantized level when updating. Using our proposed network size to implement 10-digits MNIST classification requires at least 199,000 × 8 = 1.5 Mbit array. Given our array size limitation of 512 kbit, we reduce the network size to 395 input and 10 output neurons to classify three classes (“0”, “3”, and “4”) from MNIST. Figure 5a shows recognition accuracy as a function of bit precision in the range of 5–12 bits, corresponding to quantization to 2^5^ and 2^12^ discrete levels. The recognition accuracy stays relatively constant down to 8 bits but shows a steep decrease for bit precisions < 7 bits. For hardware implementation of online unsupervised learning, the weights are updated on the subquantum CBRAM array at run-time. Figure 5b shows experimentally obtained weight maps from the subquantum CBRAM array for the 10 output neurons for the no pruning and 50% soft-pruning cases after unsupervised online training with 1000 MNIST samples. Weight update history during the online training process is investigated. Supplementary Figures 10a and b show the number of switching cycles of every bit in CBRAM cells for no pruning and 50% soft-pruning, respectively. Least significant bits (LSB) update more frequently than the most significant bits (MSB) in both cases. For the no pruning case, all bits are constantly updated throughout training, causing extensive energy consumption through programming and erasing of the subquantum CBRAM devices. In contrast, pruning reduces the number of switching cycles for all of the individual bits and the number of cumulative switching cycles as shown in Supplementary Figure 10b and Supplementary Figure 10c, respectively. Figure 5c shows the accuracy for the pruning and no pruning cases for the experimental results obtained with the subquantum CBRAM array as a function of training set size. This hardware implementation achieves 93.19% accuracy, which is very close to the accuracy for no pruning (93.68%) and the 8-bit and 64-bit ideal software implementations. Figure 5d shows the number of bit updates by device updates vs. training set size, where the data for the first 1000 samples are obtained from the hardware implementation, and the rest is computed using software simulations. The number of bit updates for both cases is identical until pruning starts. After all output neurons are pruned, the 50% pruned network has around twofold reduction in the number of bit updates compared to the no pruning case. Although our hardware demonstration focuses on 50% pruning, our simulations suggest that pruning percentages up to 80% can be implemented to further increase energy savings.

The performance of our hardware implementation for unsupervised learning is far superior to the previous state-of-the-art unsupervised learning of MNIST dataset with synaptic devices in terms of recognition accuracy, energy consumption per programming, number of weight updates in training, and network size (Supplementary Table 3). For energy consumption per programming event, subquantum CBRAM is two to three orders of magnitude more efficient than transistor-based devices (Supplementary Table 1) and shows the lowest energy consumption among RRAM based synaptic devices (Supplementary Table 3). Our pruning algorithm can reduce the number of parameter updates significantly and lead to ~20× less number of parameter updates compared to previous reports (Supplementary Table 3). Combining device level energy savings provided by subquantum CBRAM with network level energy savings by pruning may lead up to two orders of magnitude reduction in total energy consumption for hardware implementation of weight updates during unsupervised learning.

Compared to other software simulations in the literature (Supplementary Table 4), our network achieves a high classification accuracy on MNIST dataset using the lowest number of neurons and synapses and a low-complexity one-layer architecture that can be easily mapped onto 1T1R or crossbar arrays. Supplementary Table 5 compares hardware demonstration of our pruning method with other software approaches of pruning in terms of energy savings and accuracy loss. Our method provides comparable energy savings with minimal accuracy loss, while being the only method, which can be applied during the training. Last but not least, our work presents the demonstration of mapping of pruning onto a hardware platform.

## Discussion

In this study, we demonstrate unsupervised learning using an energy efficient subquantum CBRAM array. Synaptic pruning is implemented during the training and mapped onto hardware to reduce energy consumption while maintaining a classification accuracy close to ideal software simulations. We show that subquantum CBRAM cells are capable of gradual and linear conductance changes desirable for implementing online training in hardware and can be directly programmable into different conductance states indicating their potential for implementing a broad range of weight update rules for neuromorphic applications. Following a software/hardware co-design approach, we develop a neuro-inspired synaptic pruning method to significantly reduce the number of parameter updates during neural network training. Low-energy subquantum CBRAM devices combined with the network-level energy savings achieved by pruning can provide a promising path toward realizing AI hardware based on spiking neural networks that can autonomously learn and handle large volumes of data. Our hardware/software co-design approach can also be adapted to other network models to reduce the energy cost in implementing network training in low-power mobile applications.

## Methods

### Neural network algorithm

Here we describe the network architecture of the SNN including the input and output layers. Then, we explain our training, labeling, and classification procedure for the MNIST dataset. Supplementary Table 6 summarizes the parameters used in simulations.

(A) Network architecture: Our SNN is a one-layer network defined by the number of inputs neurons *m*, the number of outputs neurons *n*, and an *m* by *n* weight matrix. Each output neuron is fully-connected to every input neuron. Our SNN has 398 input and 500 output neurons. Our output neurons do not have refractory periods and there is no lateral inhibition between them.

(B) Input layer: We crop each training sample by removing pixels that represent the background in at least 95% of the training samples. Because the pixels have intensity values in the range [0, 1], those with a value of 0 correspond to the background and are thus candidates for removal. After this step, we have 397 input neurons in total by including an additional bias term, which has an input value of 1. The weights associated with this bias input neuron are learned via the same learning rule as the other weights. Each input neuron generates a Poisson spike train *X*_*i*_ whose mean firing rate is determined linearly by the pixel intensity, where a pixel of value 0 corresponds to 0 Hz and a pixel of value 1 leads to 200 Hz. The timing of each spike that is generated by the Poisson process is rounded toward the nearest millisecond, which is the time step of the simulation.

(C) Output layer: The SNN fires an output spike from any given output neuron according to a Poisson process with the specified frequency. The output neuron that fires is chosen from a softmax distribution of the output neurons’ membrane potentials as (1):^52^

1 $$P\left( {u_k} \right) = \frac{{e^{u_k}}}{{\mathop {\sum}\nolimits_{k = 1}^N {e^{u_k}} }}$$

where *P*(*u*_*k*_) is the softmax probability distribution of the membrane potentials *u*_*k*_ (*k* = 1, …, *N*). *N* is the number of output neurons. We calculate membrane potentials *u*_*k*_ using (2)

2 $$u_k = \mathop {\sum}\limits_i {W_{ki}X_i} + b_k$$

*W*_*ki*_ is the weight between input neuron *i* and output neuron *k*. *X*_*i*_ is the spike train generated by input neuron *i* and *b*_*k*_ is the weight of the bias term.

(D) Training: The SNN displays each input sample for the first 40 ms of a 50 ms presentation period, and thus the input spikes for a given sample only occurs in this 40 ms window. Figure 3a shows an example of the input spiking activity for the duration of four training samples. We use the whole training set, which contains 60,000 samples, and train for three epochs. It is important to note that 50 ms is a virtual simulation parameter along with the firing frequency chosen for generating input spikes. In the real hardware implementation, the presentation time of one image can be much shorter than 50 ms as long as enough number of input spikes are generated. The weights are updated via STDP rule shown in Fig. 3b. The LTP and LTD rules are detailed in equation (3) and (4), respectively,

3 $${\mathrm{\Delta}} W_{{\mathrm{LTP}}} = a \times e^{ - b\left( {W + 1} \right)}$$

where *a* and *b* are parameters that control the scale of the exponential, and *W* is the current weight value. The result Δ*W* is the amount of weight update of LTP and it is dependent on current *W*. LTD is a constant depression in terms of *c* in Eq. (4),

4 $${\mathrm{\Delta}} W_{{\mathrm{LTD}}} = - c$$

(E) Labeling: After training is done, we fix the trained weights and assign a class to each neuron by the following steps. First, we present the whole training set to the SNN and record the cumulative number of output spikes *N*_*ij*_, where *i* = 1,…, *N* (*N* is number of output neurons) and *j* = 1, …, *M* (*M* is number of classes). Then, for each output neuron *i*, we calculate its response probability *Z*_*ij*_ to each class *j* using Eq. (5). Finally, each neuron *i* is assigned to the class that gives the highest response probability *Z*_*ij*_.

5 $$Z_{ij} = \frac{{N_{ij}}}{{\mathop {\sum}\nolimits_{j = 1}^M {N_{ij}} }}$$

(F) Classification: We use the standard test set which contains 10,000 images. We use equation (6) to predict the class of each sample, where *S*_*jk*_ is the number of spikes for the *k*th output neuron that are labeled as class *j* and *N*_*j*_ is the number of output neurons labeled as class *j*^53^.

6 $$J = \mathop {{argmax}}\limits_j \frac{{\mathop {\sum}\nolimits_{k = 1}^{N_j} {S_{jk}} }}{{N_j}}$$

(G) Weight mapping for analog synapse implementation: The network weights (W) ranging from −1 to 1 are mapped to the device conductance data range from ~1 to 200 µS, we map the device conductance to the weight range [−1, 1] by using below linear transformation (7),

7 $$G_{{\mathrm{NORM}}} = \frac{{G - \frac{{G_{{\mathrm{max}}} + G_{{\mathrm{min}}}}}{2}}}{{\frac{{G_{{\mathrm{max}}} - G_{{\mathrm{min}}}}}{2}}}$$

In Eq. (7), we denote this normalized conductance as *G*_NORM_*. G, G*_max_, and *G*_min_ are extracted from experimental data (Fig. 2).

### Hardware Implementation

For the hardware demonstration of unsupervised learning and pruning shown in Fig. 5. CBRAM devices are employed as binary synapses. The network contains 395 input neurons (crop using the same method explained in '(B) Input layer') and 10 output neurons to classify three classes from MNIST. In 3-digits classification, out of the ~20,000 samples that represent the digits “0”, “3”, or “4” in the entire MNIST dataset, we randomly sample 5000 to create our training set. We present this training set for one epoch to train our SNN. We form the test set by drawing 10,000 samples from the remaining 15,000 samples. Neurons are implemented using a custom software to program the digital peripheral circuitry of the chip. Weight summation is performed by this program to implement the integrate-and-fire neuron. Weight update values are converted into programming pulses by the peripheral circuitry to update binary weights in the digital implementation. Fixed wordline voltages are used for binary programming of CBRAM devices. We use 8 bits to represent a synaptic weight in the network, where 1 bit is used to represent the sign of the weight value and the other 7 bits stores the absolute weight value. Bit 1 is MSB and bit 7 is LSB. The weight range [−1,1] is first uniformly divided into 256 (2^8^) discrete intervals $\left[ { - 1} \right. + \frac{i}{{128}}, - 1 + \left. {\frac{{i + 1}}{{128}}} \right)$, where *i* = 0, …, 255. Then we map the weight whose value lies in the *i*th interval to the *i*th discrete values. For example, the weights between [−1, −0.9921875) are mapped to 00000000, whereas the weights between [−0.9921875, −0.984375) are mapped to 00000001, etc. For the boundary case where the weight takes the value of 1, we map it to 11111111. The weights are updated on the hardware at run-time. We track the weight update history during the online training process (Supplementary Figure 10).

### Code availability

The code that used for the software simulation for this study are available from the corresponding authors upon reasonable request.

## Electronic supplementary material

Supplementary Information

Description of Additional Supplementary Files

Supplementary Movie 1

Supplementary Movie 2

## Data Availability

The data that support the findings of this study are available from the corresponding authors upon reasonable request.
