# Supplementary material for: Neuroinspired unsupervised learning and pruning with subquantum CBRAM arrays
Source: Nat Commun. 2018 Dec 14;9:5312. doi: 10.1038/s41467-018-07682-0 (PMC6294253; doi:10.1038/s41467-018-07682-0)
Supplement: Supplementary file 1 — Supplementary Information [file 41467_2018_7682_MOESM1_ESM.pdf]

Neuroinspired Unsupervised Learning and Pruning with Subquantum CBRAM Arrays

Supplementary Information

Shi et al.

-Supplementary Figures

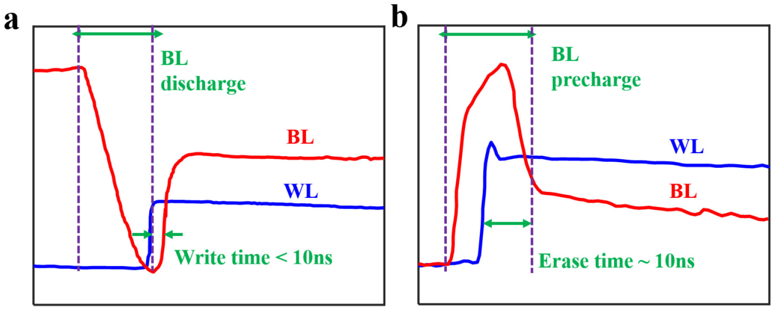

Supplementary Figure 1 | CBRAM Write/Erase Speed. a, Bitline (BL) and wordline (WL) during a 3V program operation. The anode voltage is fixed at the 3V BL voltage. After the WL is enabled, the cell programs in <10ns. **b,** Bitline (BL) and wordline (WL) during an erase operation. After the WL is enabled, the cell erases in ~10ns[1]. For programming, the voltage to be applied to the cell is established when the BL discharges (red curve). After that, the WL (blue curve) is enabled. When the cell programs, the BL voltage increases towards the anode voltage (which is high). The programming time is the offset between the time when the WL is enabled and the time when the BL voltage is seen to increase, which is shown to be <10 ns in (a). The situation is similar for erase operation, where only the polarity is reversed (BL is high). The erase time is the offset between the time when WL is enabled and the time when the BL pulls down towards the anode (which is low). Erase time is measured as ~10ns as seen in (b).

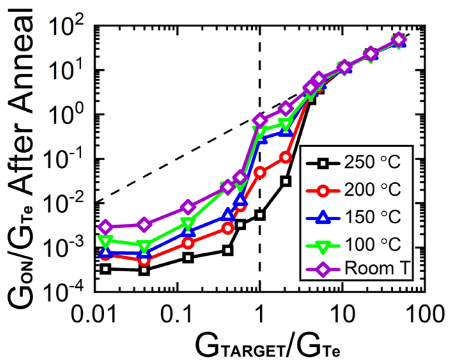

Supplementary Figure 2 | CBRAM Retention Characteristic. Excellent retention is achieved by the subquantum cells for 10 min annealing at high temperature with the ON-state conductance a few times greater than G_{Te} are targeted[2].

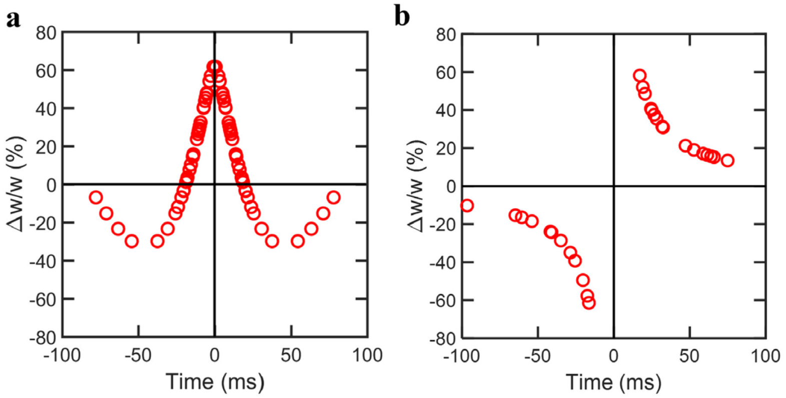

Supplementary Figure 3 | STDP. a, Symmetric spike-timing-dependent plasticity (STDP) and **b,** Asymmetric STDP learning rules modeled using the gradual programming data of 1T1R subquantum CBRAM cells in **Fig 2a.**

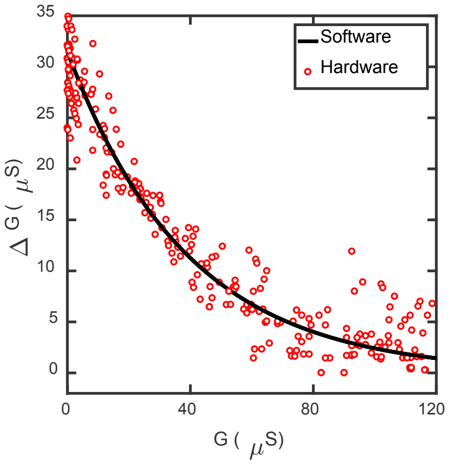

Supplementary Figure 4 | STDP Fitting. The measured data from **Fig 2b** is fitted into the neural network weight updating rule (**Fig. 3c**).

Algorithm 1: Unsupervised SNN Training

Input: Training digits
Initialization: STDP parameters, synaptic weights
for Each simulation time step t = 1ms **do**
- **STEP 1** Present a training sample for 50ms and generate input Poisson spike train X_i from 0 – 200 Hz according to pixel intensity P_i
- **STEP 2** Compute membrane potential for each output neuron
  $U_k(t) = \sum_i W_{ki} X_i(t) + b_k$
  $X_i(t) = 1$ **if** X_i fired within past 10 ms; **else** $X_i(t) = 0$
- **STEP 3** Generate output Poisson spike train via probabilistic firing model
- **STEP 4** Update corresponding neuron's weights using STDP
  $W_{ki} = W_{ki} + \Delta W_{ki}$
  ΔW_{ki} is updated via LTP rule **if** X_i fired within past $\sigma = 10$ ms;
  else ΔW_{ki} is updated via LTD rule

end for

Supplementary Figure 5 | Unsupervised Learning Algorithm. Unsupervised spiking neural network (SNN) learning algorithm used in software neural network simulation and hardware demonstration.

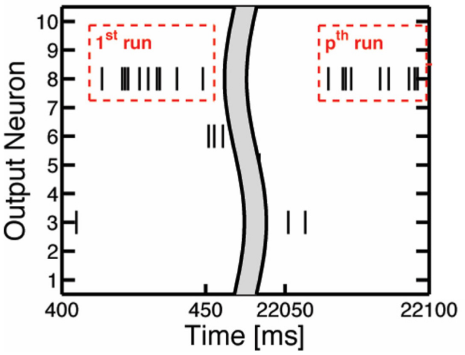

Supplementary Figure 6 | Consecutive Spikes. The illustration of consecutive output spikes of 10 output neurons as a representative example. The consecutive output spikes of Neuron 8 are boxed in red. The consecutive spikes can be measured using integrate-and-fire neuron circuits which contain capacitors[3] or memristor[4] to store the information about how many spikes they received within a time interval representing using charge or resistance.

Algorithm 2: Soft-pruning during unsupervised learning

Input: pruning percentage **r**, valid runs **p**, spike count **c**, number of weights **N**
  if there is an output spike **then**
    if this neuron has yet to be pruned **then**
      STEP 1 Count **p** for each neuron
      STEP 2 Check each sample present time window
        1. if an output neuron has accumulated **p** runs
        2. if each of those run has at least **c** consecutive output spikes
      STEP 3 If both criteria in **STEP 2** are met
        1. Set the weight threshold to the pre-determined threshold which represents $\approx$ **r**th percentile of AAthe neuron's weights
        2. Set all weights below the threshold to the lowest possible values $W_{min} = -1$.
    end if
  end if

Supplementary Figure 7 | Pruning Algorithm. Soft-pruning during the training algorithm for hardware implementation.

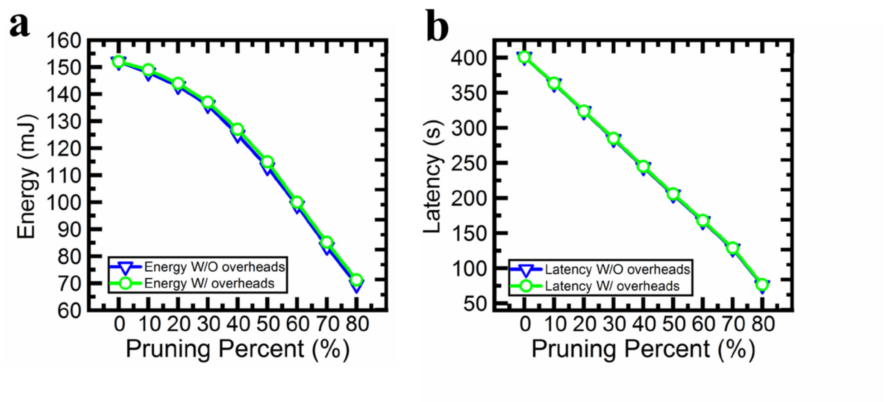

Supplementary Figure 8 | Pruning Overheads. a, Energy and **b,** Latency without and with overheads estimation for soft-pruning from 10% to 80% with a step of 10% using SNN+NeuroSim. Overheads include hardware flag and setting pruned weights to -1. Without overheads (W/O overheads) results mean that flagging mechanism is implemented in software and overhead associated

with setting pruned weights to -1 is not considered. With overheads (W/ overheads) results mean that flagging mechanism is implemented in hardware and overhead associated with setting pruned weights to -1 is considered.

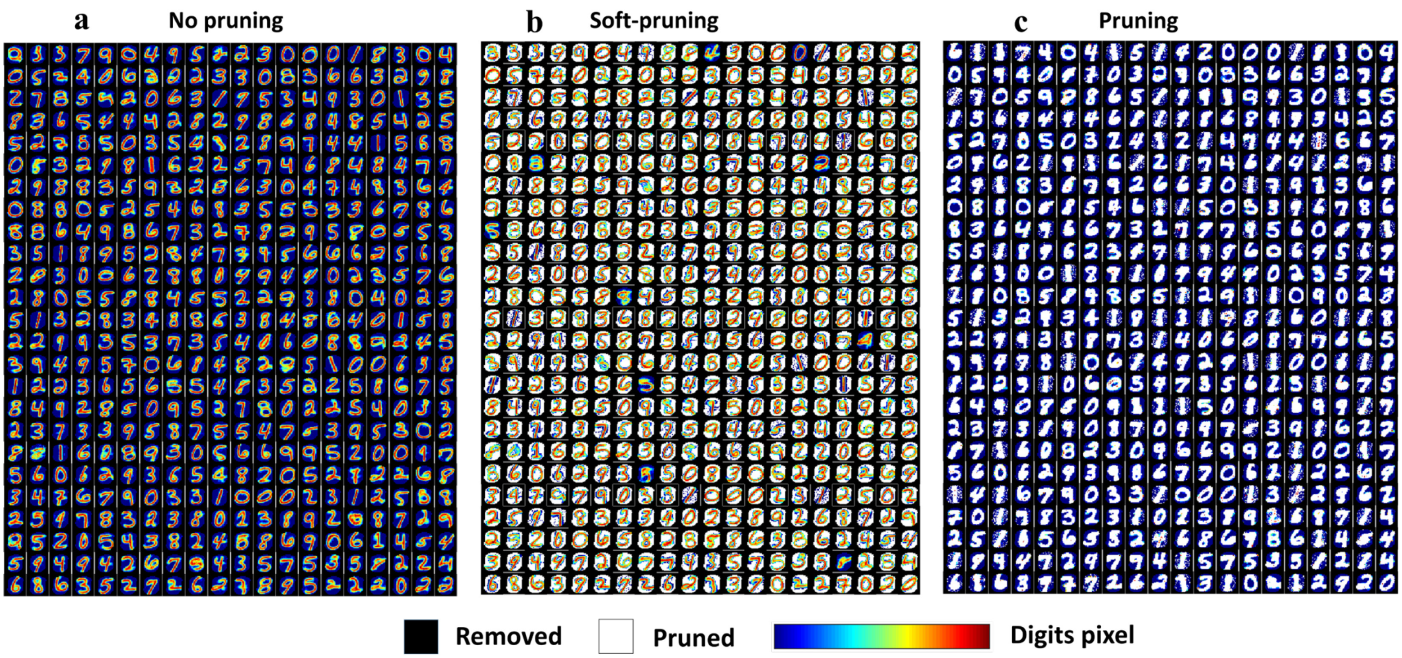

Supplementary Figure 9 | Classification and Pruning Visualization. Weights visualization of all 500 output neurons for **a,** no pruning, **b,** 50% soft-pruning and **c,** pruning after training.

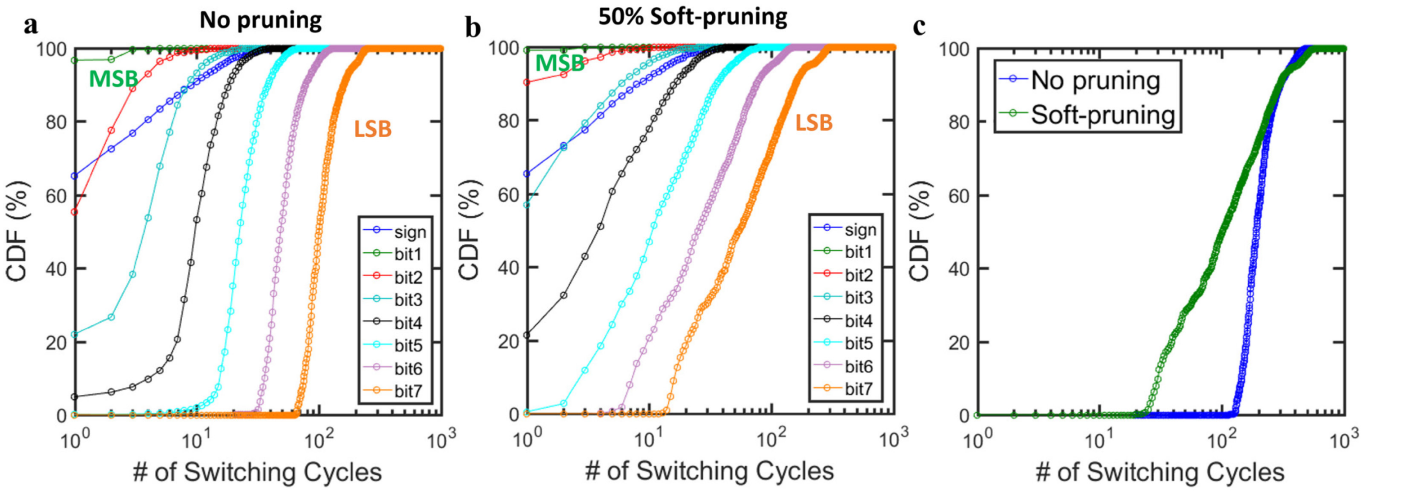

Supplementary Figure 10 | Device Switching Cycles during Training. a, b, Empirical cumulative distribution of the switching cycles of each bit in the weight matrix during training (a) no pruning and (b) with 50% soft-pruning. We use one bit for the sign. Bit 1 is MSB and bit 7 is LSB. LSB updates more frequently than MSB in both cases. 50% pruning method effectively reduces the weight updates in every bit. **c,** Cumulative distribution of the switching cycles of all bits. Pruning significantly reduces the number of switching cycles for all the bits during training.

-Supplementary Tables

Supplementary Table 1 Device Energy Profile

	Subquantum CBRAM Synapses	Metal Filament CBRAM Synapses	Floating Gate Flash
**G_{1atom}**	0.03 G_0	1 G_0	-
**Read voltage**	1V (for WL)	1V (for WL)	3-5 V (for WL)
**Program voltage**	1–3 V	1–3 V	|6| – |9| V
**Program time**	0.01 – 0.1 μs/cell	0.01 – 0.1 μs/cell	1 – 10 μs/cell
**Program energy**	0.1 – 10 pJ/cell	1-100 pJ/cell	1000 pJ/cell
**Erase voltage**	1-3 V	1-3 V	|6| – |9| V
**Erase time**	0.01 – 0.1 μs/cell	0.01 – 0.1 μs/cell	1 ms/cell
**Erase energy**	0.1 – 10 pJ/cell	1-100 pJ/cell	1000 pJ/cell

Supplementary Table 1 | Device Energy Profile. Energy consumption in subquantum conductive bridging RAM (CBRAM), metal filament-based CBRAM cells and floating gate flash[5]. Subquantum CBRAM is 10× more energy efficient than metal filament CBRAM and 100× more energy efficient than floating gate flash, even for the maximum energy consumption cases.

Supplementary Table 2 Pruning Overheads Estimation

**Accuracy (%)**	**No Pruning (8-bit)**	**80% soft-Pruning W/O overheads (8-bit)**	**80% soft-Pruning W/ overheads* (8-bit + 1-bit flag)**
**Area (μm^2)**	47233.8	47233.8	53218.5
**Energy (mJ)**	151.9	69.5	71.3 (+1.09[a], +0.68[b])
**Latency (s)**	401.1	75.6	76.5 (+0.42[a],+0.50[b])

* Overheads include [a] hardware flag and [b] setting pruned weights to -1.

Supplementary Table 2 | Pruning Overheads Estimation. Area, energy and latency estimation of no pruning, 80% soft-pruning without and with overheads. Without overheads (W/O overheads) results mean that flagging mechanism is implemented in software and overhead associated with setting pruned weights to -1 is not taken into account. With overheads (W/ overheads) results mean that flagging mechanism is implemented in hardware and overhead associated with setting pruned weights to -1 is taken into account. The numbers inside of the parentheses show the energy and latency increase due to overheads associated with [a]hardware flag and [b]setting pruned weights to -1, respectively.

Supplementary Table 3 State-of-the-Art Unsupervised Learning Demonstration with Synaptic Devices on MNIST

Architecture	Preprocessing	Learning-rule	# Neurons/ # Plastic synapses	Performance	Hardware Type	Energy Consumption	# of Updates (10^6)
Two layer network[6,7,8]	None	Exponential STDP	1,184/313,600	91.6%	HfO*x* based RRAM (simulation)	~0.85 – 24 pJ	~141
One layer network[9,10]	None	Exponential STDP	1,084/235,200	87.4%	STT-MRAM (simulation)	~0.09 – 96.9 pJ	~47
One layer network [11,12]	None	Probabilistic prespike rule	834/39,200	84%	WO*x* based RRAM (simulation)	~1.68 nJ	~47
One layer network[13]	None	Exponential STDP	864/62,720	70%	CNT synaptic transistors (simulation)	~40 – 800 nJ	~47
One layer network[14]	None	Exponential STDP	794/7,840	60%	CNT synaptic transistors (simulation)	~50 nJ	~47
One layer network[15]	yes	Exponential STDP	206/~1,980	59.8%	TiO*x* based RRAM (simulation)	~25 – 750 nJ	~8.8
**This work**	**yes**	**Exponential STDP**	**405/3,950**	**93.19%**	**Subquantum CBRAM (Hardware implementation)**	**~0.1 – 10 pJ**	**No prune: ~4.2 50% prune: ~2**

Supplementary Table 3 | Supplementary Table 3 compares the overall performance of this work with the state-of-the-art unsupervised learning demonstration with synaptic device on MNIST dataset. All the references report recognition performance simulated using single device data, while this work reports recognition accuracy for hardware implementation. The synaptic device energy consumption per programming is calculated by multiplying the pulse amplitude with the current flowing across the device and the programming pulse width. The number of updates is calculated by multiplying number of iterations in training with number of weights needed to be updated per iterations. The numbers of neurons are counted by summing up input and output neurons. If the first cell of a row contains multiple citations, subsequent values may have been taken from any one of the cited works, which are written or cited by the same authors.

Supplementary Table 4 State-of-the-Art Software Demonstration of Unsupervised Learning Demonstration on MNIST

Architecture	Preprocessing	Learning-rule	# Neurons/ # Plastic synapses	Performance	Hardware Type
Spiking Deep neural network[16]	None	Simplified STDP	N/A	98.4%	No
Two layer network[8]	None	Exponential STDP	7,184/5,017,600	95%	No
Two layer network[17]	yes	Exponential STDP	~600/~50,000	80.14%	No
**This work**	**yes**	**Exponential STDP**	**~898/~199,000**	**94.05%**	**Subquantum CBRAM**

Supplementary Table 4 | Supplementary Table 4 compares the performance (recognition accuracy) of this work with the state-of-the-art software demonstrations of unsupervised learning on MNIST dataset. The numbers of neurons are counted by summing up input and output neurons.

Supplementary Table 5 State-of-the-Art Pruning Techniques

Pruning Method	Machine Learning Tasks	Network Structure	During Training	After Training	Hardware Implementation of Pruning	Simulation System	Energy Savings	Accuracy Loss (50% pruning)
Deep Compression[18]	ImageNet pattern recognition	AlexNet	no	yes	no	EIE[22] (SRAM)	53%	~ -0.1% – +0.1%
SIMD-Aware Pruning[19]	ImageNet pattern recognition	AlexNet	no	yes	no	CPU,GPU	~53%	~ -1% – +1%
Energy-Aware Pruning[20]	ImageNet pattern recognition	AlexNet	no	yes	no	CPU	73%	< 1%
Structured Pruning[21]	CIFAR10 pattern recognition	CNN	no	yes	no	CPU	N/A	~ -1% – +2%
**This work**	**MNIST pattern recognition**	**Fully Connected**	**yes**	**yes**	**yes**	**CBRAM synaptic array**	**54.2%**	**During: +0.9%**<br>**After: -0.5%**

Supplementary Table 5 | Pruning Techniques. This table summarizes pruning methods. The first four can only be applied after training as reported by the references. The method described in this work is the first to be implemented in hardware and also can be applied either during or after training. Energy savings are relative to the result with no pruning. Energy savings for this work is obtained from **Supplementary Table 2**. Accuracy loss is based on the result with 50% pruning.

Supplementary Table 6 Simulation Parameters

Parameters		10-Digits		
		Training	Labeling	Testing
# of Neuron	Input	398		
	Output	500		
Firing Rate (Hz)	Input	200	200	200
	Output	200	200	600
Image Presenting Time (ms)		50	50	200
Pruning Threshold	Prune Parameter (p)	10	-	-
	Spike Count	8	-	-
STDP		a = 0.0667<br>b = 2.5<br>c = 0.0167		

Supplementary Table 6 | Simulation Parameters. All the parameters used for the simulations are listed above.

-Supplementary Notes

Supplementary Note 1

Supplementary Figure 2 shows an experimentally quantified stability (retention) as a function of conductance. It can be seen that the subquantum CBRAM has robust thermal stability. In this figure, the x-axis is the target cell conductance during a program operation. Note that the x-axis is normalized so that the value 1 corresponds to the conductance of a filament with a 1-atom constriction. The y-axis is the actual conductance measured after annealing at temperatures ranging from room temperature to 250 °C. The plot shows that stability (retention) is poor if the targeted conductance level is lower than the conductance of an incomplete filament whose thinnest spot is less than 1 atom thick. However, once the target conductance is above the value of the 1-atom thick filament, the post-anneal conductance quickly approaches the targeted value. Therefore, the filament is increasingly stable as it becomes thicker. Note that filaments can be stable even at the highest temperature used in the study (250 °C).

Supplementary Note 2

Asymmetric and symmetric STDP are implemented using the same spike scheme described by *Kuzum. et al.* [23]. Pre and post spikes are implemented to the word line and bit line of the 1T1R array. Time overlap of the pre and post spikes allows programming of the CBRAM synapse. The spike timing differences between the pre and post spikes are translated to the amplitude of voltage pulses applied to the word line. Integrate-and-fire neurons are implemented using a computer program and pulse generators, and the pulses are applied to the WL and BL of the device to modulate the conductance change.

Supplementary Note 3

The overhead costs of the pruning algorithm can be estimated using our SNN platform for NeuroSim. The first overhead is that the pruned weights need to be flagged to prevent them from further updating. This can be implemented by adding an additional bit with an initial value of 0 to serve as a hardware flag for pruning. We update the pruning flags of an output neuron's weights to '1' when they have been pruned during the training. Note that since the weights are only pruned once during the entire training, each hardware flag is just written once. Before weight update, we read the hardware flag of the winner neuron's weights and the weight will not be updated if its flag is '1'. Another overhead is setting the pruned weights to -1. We take these two overheads into account in our simulation for pruning using digital hardware implementation (**Fig.2c**). In **Supplementary Table 2**, both overhead costs are estimated in terms of area, energy and latency based on the peripheral programming circuitry shown in Fig. 2c for no pruning, 80% soft-pruning without (W/O overheads) and with overheads (W/ overheads). As can be seen from this table, the area is increased by ~12.7% because the flag only takes up one extra bit for each synapse. The hardware flag increases energy and latency by ~1.6% (1.09mJ) and ~0.6% (0.42s), respectively. Setting pruned weights to -1 increases energy and latency by ~0.98% (0.68mJ) and ~0.66% (0.5s), respectively. In summary, total energy and latency are increased by ~2.5% (~1.7mJ) and ~1.2% (~0.92s) due to the

overheads of pruning implementation. This is significantly smaller and hence negligible compared to the energy and latency gains from pruning.

-Supplementary References

1 Jameson, J. R. *et al.* Conductive-bridge memory (CBRAM) with excellent high-temperature retention. In *IEEE International Electron Devices Meeting (IEDM).* 30.31.31-30.31.34 (2013).

2 Jameson, J. R. &Kamalanathan, D. Subquantum conductive-bridge memory. *Applied Physics Letters* **108**, 053505 (2016).

3 Indiveri, G. A low-power adaptive integrate-and-fire neuron circuit. In *Proceedings of the 2003 International Symposium on Circuits and Systems, 2003. ISCAS '03.* IV-IV (2003).

4 Zhang, X. *et al.* An Artificial Neuron Based on a Threshold Switching Memristor. *IEEE Electron Device Letters* **39**, 308-311 (2018).

5 Jameson, J. R. *et al.* Conductive bridging RAM (CBRAM): then, now, and tomorrow. *ECS Transactions* **75**, 41-54 (2016).

6 Tosson, A. M. S., Yu, S., Anis, M. H. &Wei, L. A Study of the Effect of RRAM Reliability Soft Errors on the Performance of RRAM-Based Neuromorphic Systems. *IEEE Transactions on Very Large Scale Integration (VLSI) Systems* **25**, 3125-3137 (2017).

7 Kuzum, D., Yu, S. &Wong, H. P. Synaptic electronics: materials, devices and applications. *Nanotechnology* **24**, 382001 (2013).

8 Diehl, P. &Cook, M. Unsupervised learning of digit recognition using spike-timing-dependent plasticity. *Frontiers in Computational Neuroscience* **9** (2015).

9 Zhang, D., Zeng, L., Zhang, Y., Zhao, W. &Klein, J. O. Stochastic spintronic device based synapses and spiking neurons for neuromorphic computation. In *IEEE/ACM International Symposium on Nanoscale Architectures (NANOARCH).* 173-178 (2016).

10 Vincent, A. F. *et al.* Spin-Transfer Torque Magnetic Memory as a Stochastic Memristive Synapse for Neuromorphic Systems. *IEEE Transactions on Biomedical Circuits and Systems* **9**, 166-174 (2015).

11 Sheridan, P., Ma, W. &Lu, W. Pattern recognition with memristor networks. In *IEEE International Symposium on Circuits and Systems (ISCAS).* 1078-1081 (2014).

12 Chang, T., Jo, S.-H., Kim, K.-H., Sheridan, P., Gaba, S. &Lu, W. Synaptic behaviors and modeling of a metal oxide memristive device. *Applied Physics A* **102**, 857-863 (2011).

13 Kim, S. *et al.* Pattern Recognition Using Carbon Nanotube Synaptic Transistors with an Adjustable Weight Update Protocol. *ACS Nano* **11**, 2814-2822 (2017).

14 Kim, S., Yoon, J., Kim, H.-D. &Choi, S.-J. Carbon Nanotube Synaptic Transistor Network for Pattern Recognition. *ACS Applied Materials & Interfaces* **7**, 25479-25486 (2015).

15 Zahari, F., Hansen, M., Mussenbrock, T., Ziegler, M. &Kohlstedt, H. Pattern recognition with TiOx-based memristive devices. *AIMS Material. Science* **2**, 203-216 (2015).

16 Kheradpisheh, S. R., Ganjtabesh, M., Thorpe, S. J. &Masquelier, T. STDP-based spiking deep convolutional neural networks for object recognition. *Neural Networks* **99**, 56-67 (2018).

17 Nessler, B., Pfeiffer, M., Buesing, L. &Maass, W. Bayesian computation emerges in generic cortical microcircuits through spike-timing-dependent plasticity. *PLoS computational biology* **9**, e1003037 (2013).

18 Han, S., Pool, J., Tran, J. &Dally, W. Learning both weights and connections for efficient neural network. In *Advances in neural information processing systems.* 1135-1143 (2015).

19 Yu, J., Lukefahr, A., Palframan, D., Dasika, G., Das, R. &Mahlke, S. Scalpel: Customizing DNN pruning to the underlying hardware parallelism. In *2017 ACM/IEEE 44th Annual International Symposium on Computer Architecture (ISCA).* 548-560 (2017).

20 Yang, T., Chen, Y. &Sze, V. Designing Energy-Efficient Convolutional Neural Networks Using Energy-Aware Pruning. In *IEEE Conference on Computer Vision and Pattern Recognition (CVPR).* 6071-6079 (2017).

21 Anwar, S., Hwang, K. &Sung, W. Structured pruning of deep convolutional neural networks. *ACM Journal on Emerging Technologies in Computing Systems (JETC)* **13**, 32 (2017).

22 Han, S. *et al.* EIE: Efficient Inference Engine on Compressed Deep Neural Network. In *ACM/IEEE 43rd Annual International Symposium on Computer Architecture (ISCA).* 243-254 (2016).

23 Kuzum, D., Jeyasingh, R. G. D., Yu, S. &Wong, H. P. Low-Energy Robust Neuromorphic Computation Using Synaptic Devices. *IEEE Transactions on Electron Devices* **59**, 3489-3494 (2012).
