# Supplementary material for: Neuroinspired unsupervised learning and pruning with subquantum CBRAM arrays
Source: Nat Commun. 2018 Dec 14;9:5312. doi: 10.1038/s41467-018-07682-0 (PMC6294253; doi:10.1038/s41467-018-07682-0)
Supplement: Supplementary file 2 — Description of Additional Supplementary Files [file 41467_2018_7682_MOESM2_ESM.pdf]

Description of Additional Supplementary Files

File Name: Supplementary Movie 1
Description: The development of the 10 representative output neurons' weights during the training for soft-pruning.

File Name: Supplementary Movie 2
Description: The development of the 10 representative output neurons' weights during the training for no pruning
